# A Novel Algorithm in the Management of Hypoglycemia in Newborns

**DOI:** 10.1155/2014/935726

**Published:** 2014-11-12

**Authors:** Swapna Naveen, Chikati Rosy, Hemasree Kandraju, Deepak Sharma, Tejopratap Oleti, Srinivas Murki

**Affiliations:** Department of Neonatology, Fernandez Hospital, Hyderguda, Hyderabad, Telangana 500029, India

## Abstract

*Study Objective*. To evaluate the safety of a new protocol in comparison to the standard protocol for managing hypoglycemia in neonates. *Methods*. Open label RCT-pilot study. Neonates admitted to NICU with hypoglycemia and requiring intravenous fluids were included. Fifty-seven eligible neonates were randomly allocated to either intervention group (starting fluids with 10% dextrose and increments of 1.5%) or standard protocol group (GIR of 6 mg/kg/min with increments of 2 mg/kg/min) till control of hypoglycemia. Primary outcome of the study was to know proportion of infants with subsequent hypoglycemia and hyperglycemia after enrolment. *Results*. The initial GIR (6 ± 0 mg/kg/min versus 4.8 ± 1.4 mg/kg/min, *P* < 0.001), the mean maximum GIR (6.7 ± 1.6 mg/kg/min versus 5.6 ± 2 mg/kg/min, *P* = 0.03), the maximum concentration of glucose infused (13.8 ± 2.9% versus 10.9 ± 1.9%, *P* < 0.001), and the total amount of glucose infused were significantly lower in the intervention group. The mean maximum blood sugar was significantly higher (129 ± 57 mg/dL versus 87 ± 30 mg/dL, *P* = 0.001) and there was a trend towards high proportion of infants with Hyperglycemia in the standard protocol group (*n* = 10, 39% versus *n* = 5, 16%, *P* = 0.07). The median difference between the highest and the lowest recorded sugar for any infant was significantly higher in the standard protocol group (median 93 mg/dL, IQR 52 to 147 mg/dL versus median 50 mg/dL, IQR 38 to 62.5 mg/dL, *P* = 0.03). *Conclusion*. A new and novel algorithm in the management of hypoglycemia in neonates is as safe as the standard protocol and requires further testing before routine implementation.

## 1. Introduction

The term “hypoglycemia” refers to a reduction in the glucose concentration of circulating blood. It is almost 100 years since hypoglycemia was first described in children and over 50 years since it was recognized in newborn and older infants [1]. It is recognized that 23–50% of infants admitted to neonatal intensive care unit are diagnosed with one or more episodes of hypoglycemia [2–4]. In an Indian study, Singh et al. reported the incidence of hypoglycemia in 9.6%, 15.3%, and 19.4% term AGA, term SGA, and term LGA infants, respectively [5]. Neonatal hypoglycemia adversely affects the neurodevelopmental outcome, overall IQ, reading ability, arithmetic proficiency, and motor performance [6]. Hence, there is a need to correct the blood sugar as early as possible. Standard protocol for correction of hypoglycemia in symptomatic newborns or when blood sugar is <30 mg/dL involves giving a bolus of 2 mL/kg of 10% dextrose followed by a glucose infusion rate of 6 mg/kg/min. The current protocol in many units is that if hypoglycemia persists, increments are made in the glucose infusion rate (GIR) at 2 mg/kg/min every 15 to 30 minutes [7, 8]. This method is very tedious and involves many calculations and hence time lag and errors in the preparation of fluid. As the morbidities due to neonatal hypoglycemia depend on the duration of hypoglycemia, we need a better method which will reduce the time lag and errors in implementation of required GIR. We did this open labeled randomized controlled pilot study to evaluate the safety of a new protocol in comparison to the standard protocol for managing hypoglycemia in neonates.

## 2. Method and Material

This randomized pilot study was conducted in a tertiary level teaching hospital of south India. Fernandez Hospital (FH) institutional review board approved the study protocol. Consent was obtained from the parents before randomization of the infant in the study. Inclusion criteria included all neonates with hypoglycemia requiring intravenous fluids. Hypoglycemia was defined as blood glucose less than 40 mg/dL. Intravenous fluids were considered if

- blood glucose was <40 mg/dL and infant is symptomatic: symptoms included lethargy, jitteriness, poor suck, and seizures,
- blood glucose was less than 30 mg/dL, or
- blood glucose was less than 40 mg/dL fifteen minutes after an oral feed.

Sick neonates (shock requiring inotropes or ventilation or oxygen or already on intravenous fluids for any other reason) with hypoglycemia and neonates with hypoglycemia not requiring intravenous fluids (hypoglycemia corrected with feeds) and those in whom consent could not be obtained were excluded. Randomization was based on a web based random number generator. Group allocation was concealed in serially numbered sealed opaque envelopes. The envelope was opened after obtaining a written consent and after entering the infant details on the outer cover of the envelope. The principal investigator opened the envelopes and randomized the babies. After allotment of babies to intervention or standard protocol group, the necessary details were entered in a predesigned case reporting form.

### 2.1. Standard Protocol

1. Glucose infusion was started with 6 mg/kg/min.
2. Glucose infusion rate (GIR) was increased by 2 mg/kg/min every 30 minutes if blood sugar continued to be below 40 mg/dL to a maximum glucose infusion rate of 14 mg/kg/min.
3. Central venous line was established if glucose concentration exceeded 12.5% for a duration of 12 hrs.
4. Blood glucose was monitored after 30 min after initiation of infusion, 2 hours of infusion, and subsequently every 6 hours if blood glucose remained >50 mg/dL. If the blood sugar was ≤50 mg/dL, monitoring was continued every 30 minutes till sugar was above 50 mg/dL.
5. If blood sugar was >50 mg/dL but less than 125 mg/dL, tapering of GIR was done after 2 values at 6-hour interval which was between 50 and 125 mg/dL. But if the blood sugar was greater than 125 mg/dL, a repeat sample was done in 2 hours and if it was still >125 mg/dL, tapering was initiated immediately.
6. For infants on 6 mg/kg/min, tapering was to the maintenance fluid if they continued to be on intravenous fluids but if feeding was initiated, tapering of intravenous fluids was at the rate of 1 mL/hour. For infants on GIR >6 mg/kg/min, tapering was done at 2 mg/kg/min every 6 hours till GIR was 6 mg/kg/min. Once GIR was 6 mg/kg/min, feeding was initiated and fluids were tapered at 1 mL/hour.

### 2.2. Intervention Group

1. Glucose infusion was started with 10% dextrose.
2. Glucose concentration was increased in steps of 1.5% (step 1 10% to 11.5% and step 2 11.5% to 13%) every 30 minutes if blood sugar continued to be <40 mg/dL. If blood sugar remained <40 mg/dL on 2 increments, then the infant was switched to standard protocol. Glucose of 11.5% was prepared by mixing 10 mL of 25% to 90 mL of 10% dextrose. For every 1.5% increase in glucose concentration, volume of 25% dextrose increased by 10 mL and that of 10% dextrose decreased by 10 mL for every 100 mL of stock solution.
3. Step 3 to step 5 was similar to that in the standard group.
4. For infants on glucose concentration >10%, tapering was by 1.5% every 6 hours. For babies on 10% glucose, tapering was to the maintenance fluids if baby continued to be on intravenous fluids and by 1 mL/hour if baby was initiated on feeds.

In both of the groups monitoring of blood sugar was as at admission, 30 minutes, 2 hours, and 6 hours. If sugar was ≤50 mg/dL, monitoring was every 30 minutes. If sugar was >125 mg/dL, monitoring was every 2 hours. In all enrolled infants a minibolus of 10% dextrose, 2 mL/kg, was given if the infant was symptomatic at enrollment or had a sugar <30 mg/dL at enrolment. No calorie supplements or glucose polymers were used for infants on enteral feeds. Volume of intravenous fluids was based on the infant's needs, based on the day of life and birth weight and also on the hydration status. Neonates exited from the study once they were on full enteral feeds for a duration of at least 12 hours.

Primary outcomes of the study included proportion of infants with hypoglycemia (defined as blood glucose < 40 mg/dL) or hyperglycemia (defined as blood glucose > 125 mg/dL) after enrolment in the trial. Secondary outcomes included mean maximum blood glucose, difference between highest and lowest blood glucose after enrolment, need for central line placements, maximum GIR, maximum percent of dextrose infused, glucose infused in gm/kg/day, time to initiate enteral feeds, time to full enteral feeds, and discharge neurosonogram.

Blood glucose was estimated by strip method (glucose oxidase method, Optium Xceed glucometer) from a venous blood sample. Low blood sugar (blood sugar < 40 mg/dL) and high blood sugar (blood sugar > 125 mg/dL) by strip method were always confirmed with a laboratory blood glucose value. Estimation of blood glucose in the laboratory was done by automated Hexokinase method.

## 3. Statistics

Comparison of outcomes between the groups was done using chi-square test for categorical variables and Student's *t*-test or Mann-Whitney *U* test for continuous variables as appropriate. A *P*  value < 0.05 was considered to be significant. No a priori sample size was estimated for the study.

## 4. Results

**Figure 1:**
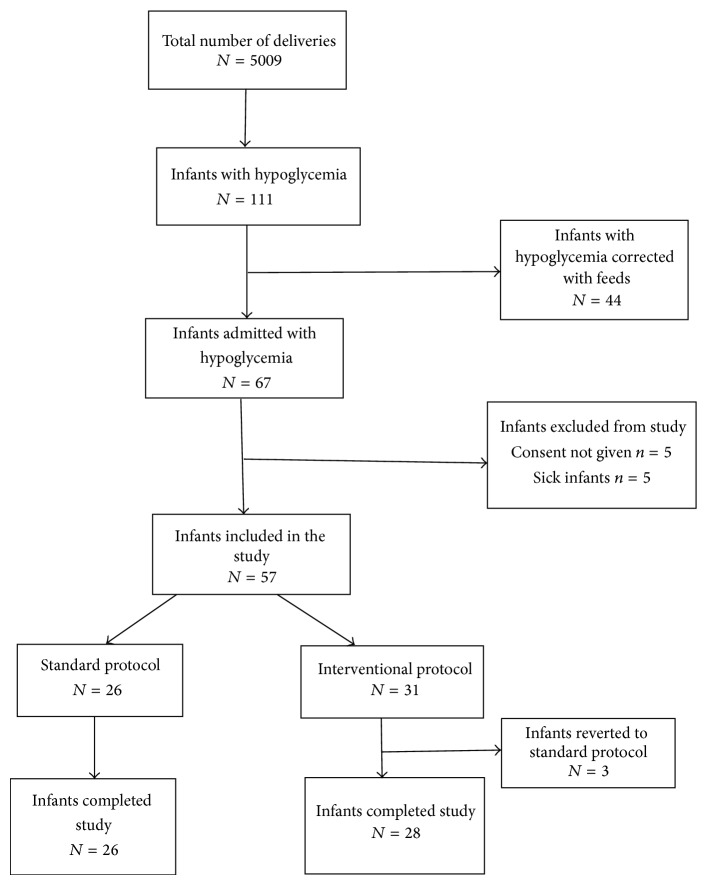
Study flow chart.

Fifty-seven infants were enrolled in the study (Figure 1). All the infants completed the study and received their allocated treatment protocols till the completion of the study. The reasons for hypoglycemia in our study group (*n* = 57) included intrauterine growth restriction *n* = 27 (47%), infant of diabetic mother *n* = 18 (31%), large for gestation *n* = 3 (5%), and prematurity *n* = 9 (17%). Both of the study groups were comparable for the baseline variables including gestational age, birth weight, maternal risk factors, intrauterine growth status, median age at enrolment, and blood sugar at enrolment (Table 1).

### 4.1. Hypoglycemia Management

Five of the 26 infants in standard protocol group had symptomatic hypoglycemia. Three infants presented with lethargy, two with jitteriness, and one each with poor feeding, irritability, and seizures. In the intervention group too, five infants had symptomatic hypoglycemia. The symptoms in the order of frequency included jitteriness (*n* = 3), lethargy (*n* = 2), poor feeding (*n* = 2), and seizure (*n* = 1). The initial glucose infusion rate (6 ± 0 mg/kg/min versus 4.8 ± 1.4 mg/kg/min, *P* < 0.001), the mean maximum glucose infusion rate (6.7 ± 1.6 mg/kg/min versus 5.6 ± 2 mg/kg/min, *P* = 0.03), the maximum concentration of glucose infused (13.8 ± 2.9% versus 10.9 ± 1.9%, *P* < 0.001), and the median total amount of glucose infused were significantly lower in the intervention group (median, IQR: 6.2 g/kg/day; 5.5 to 7.9 g/kg/day versus 5.1 g/kg/day; 4.2 to 6.2 g/kg/day, *P* = 0.005). The proportion of infants requiring increments in glucose (GIR or percent dextrose) was similar between the two groups (*n* = 5, 19% versus *n* = 8, 26%, *P* = 0.35). In the intervention group three infants required increments in glucose concentration once (to 11.5%), two infants required increments in glucose concentration twice (to 13%), and three infants required increments in glucose concentration more than twice. All three infants requiring more than two increments in glucose concentration were switched to standard protocol at GIR 2 mg/kg/min higher. Two, two, and one infants in the standard protocol group required once (8 mg/kg/min), twice (10 mg/mg/min), and more than twice (>12 mg/kg/min) increments in GIR. The number of infants requiring central line placement (*n* = 6, 23% versus *n* = 3, 9.7%, *P* = 0.27) was similar between the standard protocol and intervention group, respectively.

### 4.2. Outcomes

The proportion of infants with hypoglycemia (*n* = 5, 19% versus *n* = 8, 26%, *P* = 0.35) and also moderate hypoglycemia (blood glucose ≤ 50 mg/gL; *n* = 14, 54% versus *n* = 14, 45%, *P* = 0.60) was similar between the two groups. The mean maximum blood sugar was significantly higher (129 ± 57 mg/dL versus 87 ± 30 mg/dL, *P* = 0.001) and there was a trend towards high proportion of infants with hyperglycemia in the standard protocol group (*n* = 10, 39% versus *n* = 5, 16%, *P* = 0.07). The median difference between the highest and the lowest recorded sugar for any infant was significantly higher in the standard protocol group (median: 93 mg/dL, IQR 52 to 147 mg/dL versus median 50 mg/dL, IQR 38 to 62.5 mg/dL, *P* = 0.03). One infant in the intervention group had MRI abnormality attributable to neonatal hypoglycemia but this infant had neither seizures nor recurrence of hypoglycemia after enrolment. One infant in the standard protocol group died of neonatal sepsis and was not related to the management of hypoglycemia. The median time to initiate enteral feeds (median, IQR; 1 day; 1–2.5 days versus 1 day; 1-2 days, *P* = 0.60) and the median time to reach full enteral feeds (median, IQR; 1 day; 1–3.5 days versus 1 day; 1-2 days, *P* = 0.62) after enrolment were similar between the two groups. All enrolled infants had a normal neurosonogram at discharge.

## 5. Discussion

This pilot study was done to assess the safety of a novel algorithm for management of hypoglycemia and it was found to be as safe as the standard protocol. The proportion of infants with subsequent hypoglycemic episodes was similar between the two groups. This new protocol is as efficacious as the standard protocol as the proportion of infants requiring increments in GIR in standard protocol group was similar to that requiring increment in dextrose concentration in the intervention group. Also the time taken for initiation of oral feeds and that needed to achieve full enteral feeds after enrolment were also similar between the two groups. It is noteworthy that efficacy was similar although infants in the intervention group received lesser GIR and lower total glucose infused per day. As per the protocol, being a pilot study, 3 infants from intervention group were switched to the standard protocol group for increasing glucose requirements.

The rate of endogenous glucose metabolism in well fasting neonates is estimated to be 4 to 6 mg/kg/min. Glucose infusions commenced at 60 mL/kg/day of 10% dextrose will provide a GIR of 4 mg/kg/min [9]. In this study, the starting mean GIR in the intervention group of 4.8 ± 1.4 mg/kg/min explains the physiological reason behind glucose stabilization in this group. For newborns with severe and symptomatic hypoglycemia, AAP recommends a minibolus of 2 mL/kg of 10% dextrose and/or starting a continuous infusion of 10% dextrose at 80 to 100 mL/kg per day. If the goal of achieving plasma glucose between 40 and 50 mg/dL is not met after 24 hours of glucose infusion, a workup for hyperinsulinemic hypoglycemia is suggested. However the guideline is silent on increasing the concentration of dextrose for hypoglycemic episodes occurring within 24 hours of starting glucose infusion [10]. Standard national guidelines from India recommend a minibolus of 2 mL/kg of 10% dextrose and starting glucose infusion at a GIR of 6 mg/kg/min. After starting the glucose infusion, for persisting hypoglycemia, an increment in GIR by 2 mg/kg/min till a maximum of 12 mg/kg/min is suggested [7, 8]. A popular manual on neonatal care recommends a minibolus followed by a glucose infusion at GIR 6 to 8 mg/kg/min. When the GIR required is >12 mg/kg/min, workup for hyperinsulinemia is suggested [11]. Thus there is no evidence or consensus on the management of hypoglycemia in newborns. This is the first study to highlight these differences and to provide some evidence for a uniform protocol.

The mean maximum blood glucose and the median difference between the lowest to highest blood glucose were significantly higher in the standard protocol group. Also there was a trend towards increased incidence of hyperglycemic episodes in the standard protocol group. These changes are explained by the 33% increase in GIR from baseline (6 to 8 to 10 mg/kg/min) in the standard protocol as against 15% increase (10 to 11.5 to 13%) in this new protocol. It is well known that hypoglycemia is associated with compensatory increased cerebral blood flows and hyperglycemia with decreased cerebral blood flow and these changes if frequent may have adverse effects on the developing brain [12–14]. There is a need to assess the long term effects of these high and fluctuating blood glucose levels as seen in the standard protocol for management of hypoglycemia. In a recent report by Vanhatalo and Tammela when 15% dextrose was compared with 20% dextrose at GIR of 8 mg/kg/min for correction of hypoglycemia, 16% of the infants had hyperglycemic (plasma glucose > 7.7 mmol/L (138 mg/dL)) episodes [15].

Several studies have shown that hypoglycemia and hyperglycemia are detrimental for both short and long term neurodevelopmental outcomes [6]. We did not assess the long term outcomes of enrolled newborns in our study.

## 6. Limitation and Merits of Study

We excluded very sick infants from our study due to ethical considerations. As the infants of intervention groups were reverted to standard protocol after 2 increments, we cannot explain the effectiveness of this protocol for treatment of prolonged, refractory, or persistent hypoglycemia. There is also a need to study the effect of this hypoglycemia correction protocol on the long term neurodevelopmental outcome of the infants.

We could not find similar studies in the literature comparing the standard protocol for correction of neonatal hypoglycemia with other formulae and protocols. Ease of preparation and lesser calculations are the main advantages of this new protocol. Lesser calculations imply lesser errors in mixing of fluids and lesser delay in starting of dextrose infusions. Further larger randomized trials are needed to test this protocol for its widespread implementation not only in otherwise healthy babies but also in sick neonates.

## Figures and Tables

**Table 1:** Comparison of baseline variables between the study groups.

Variables	Standard protocol (*N* = 26); *n* (%)	Intervention protocol (*N* = 31); *n* (%)	*P* value
Gestation (mean ± SD)	2048 ± 513	2195 ± 877	0.45
Birth weight (g) (mean ± SD)	36.23 ± 2.3	36.03 ± 2.5	0.76
Male sex	18 (69)	24 (77)	0.55
GDM	8 (31)	10 (32)	1.00
1 min Apgar (median, IQR)	7 (7-8)	7 (7-8)	0.81
IUGR	14 (54)	13 (42)	0.43
Blood sugar at enrolment	27.7 ± 6.4	27.2 ± 7.3	0.42
Age at enrolment in hrs (median, IQR)	2 hrs (2 hrs–8.25 hrs)	2 hrs (1 hr–10 hrs)	0.42
